# Central chronic apelin infusion decreases energy expenditure and thermogenesis in mice

**DOI:** 10.1038/srep31849

**Published:** 2016-08-23

**Authors:** Anne Drougard, Audren Fournel, Alysson Marlin, Etienne Meunier, Anne Abot, Tereza Bautzova, Thibaut Duparc, Katie Louche, Aurelie Batut, Alexandre Lucas, Sophie Le-Gonidec, Jean Lesage, Xavier Fioramonti, Cedric Moro, Philippe Valet, Patrice D. Cani, Claude Knauf

**Affiliations:** 1 Institut National de la Santé et de la Recherche Médicale (INSERM), U1048, Université Paul Sabatier, UPS, Institut des Maladies Métaboliques et Cardiovasculaires (I2MC), CHU Rangueil, 1 Avenue Jean Poulhès, BP84225, 31432 Toulouse Cedex 4, France; 2 NeuroMicrobiota, European Associated Laboratory, (EAL) INSERM/UCL, INSERM U1220, Institut de Recherche en Santé Digestive (IRSD), CHU Purpan - Place du Docteur Baylac, CS 60039, 31024 Toulouse Cedex 3, France; 3 Focal Area Infection Biology, Biozentrum, University of Basel, Klingelbergstrasse 50/70 CH-4056 Basel, Switzerland; 4 Université catholique de Louvain (UCL), Louvain Drug Research Institute, LDRI, Metabolism and Nutrition research group, WELBIO, WELBIO (Walloon Excellence in Life sciences and BIOtechnology), Av. E. Mounier, 73 B1.73.11, B-1200, Brussels, Belgium; 5 NeuroMicrobiota, European Associated Laboratory, (EAL) INSERM/UCLAv. E. Mounier, 73 B1.73.11, B-1200, Brussels, Belgium; 6 Université de Lille, Unité environnement périnatal et santé, EA 4489, Équipe malnutrition maternelle et programmation des maladies métaboliques, Université de Lille1, Bâtiment SN4, 59655 Villeneuve d’Ascq, France; 7 Centre des Sciences du Goût et de l’Alimentation, CNRS, INRA, Univ. Bourgogne Franche-Comté, F-21000 Dijon, France

## Abstract

Apelin is a bioactive peptide involved in the control of energy metabolism. In the hypothalamus, chronic exposure to high levels of apelin is associated with an increase in hepatic glucose production, and then contributes to the onset of type 2 diabetes. However, the molecular mechanisms behind deleterious effects of chronic apelin in the brain and consequences on energy expenditure and thermogenesis are currently unknown. We aimed to evaluate the effects of chronic intracerebroventricular (icv) infusion of apelin in normal mice on hypothalamic inflammatory gene expression, energy expenditure, thermogenesis and brown adipose tissue functions. We have shown that chronic icv infusion of apelin increases the expression of pro-inflammatory factors in the hypothalamus associated with an increase in plasma interleukin-1 beta. In parallel, mice infused with icv apelin exhibit a significant lower energy expenditure coupled to a decrease in PGC1alpha, PRDM16 and UCP1 expression in brown adipose tissue which could explain the alteration of thermogenesis in these mice. These data provide compelling evidence that central apelin contributes to the development of type 2 diabetes by altering energy expenditure, thermogenesis and fat browning.

Obesity and metabolic diseases are becoming worldwide epidemics, and are considered as public health issue. Obesity is associated with low-grade chronic inflammation that contributes to the development of insulin resistance, type 2 diabetes (T2D) and cardiovascular diseases^1^. However, the mechanisms underlying diabetes, obesity, fat mass development and the onset of inflammation have not been fully defined. There are several potential mechanisms through which molecular signals have been linked to inflammatory signaling, such as mitochondrial dysfunction, oxidative stress and endoplasmic reticulum (ER) stress^2^. The brain, and more precisely the hypothalamus, has been shown to be a primary site for body dysfunction in metabolic syndrome^3^. Consequently, altered hypothalamic responses have been associated with dysfunction of metabolic and energy homeostasis, including food intake, energy expenditure^4^, nutrient sensing and glucose metabolism^3^. Moreover, the hypothalamus is the target of numerous pro-inflammatory stimuli^5^, leading to diabetes features^6^,^7^,^8^.

Apelin is a bioactive peptide identified in 1998 as the endogenous ligand of the orphan G-protein coupled receptor APJ^9^. APJ is expressed in many peripheral tissues, as well as in different brain areas including the hypothalamus^10^. In periphery, intravenous injection of apelin improves insulin sensitivity in normal and obese/diabetic mice^11^. In the brain, apelin is now considered as a new hypothalamic actor that might contribute to the control of glucose metabolism^12^,^13^,^14^.

Evidence shows a controversial role of apelin in the regulation of food intake^15^,^16^,^17^, but a consensus exists regarding its impact in the control of glucose homeostasis^12^,^13^. Although low quantity of central apelin improves fed glycaemia in normal mice, a pathophysiological increase in apelin expression in the hypothalamus generates fasting hyperglycaemia^13^. More precisely, acute and chronic intracerebroventricular (icv) infusions of apelin, that mimic the levels of apelin present in the hypothalamus of obese/diabetic mice^18^, induce T2D characteristics in normal mice, including fasting hyperglycaemia, hyperinsulinaemia and insulin intolerance^13^.

Adipose tissue is an important regulator of energy balance which plays a complex, but a key role in the control of metabolic functions. Although white adipose tissue provides lipids as energetic substrates during fasting periods, brown adipose tissue (BAT) and beiging/browning processes contribute to dissipate the chemical energy stored as heat to preserve core temperature during hypothermia and to counteract obesity^19^. BAT adipocytes contain a high number of mitochondria, which are characterized by the presence of uncoupling protein-1 (UCP1) in the inner mitochondrial membrane. After its activation, this protein uncouples the mitochondrial respiration from ATP synthesis that results in heat production corresponding to the “non-shivering thermogenesis”.

The role of BAT activation appears to be broader than solely the promotion of negative energy balance. Indeed, BAT is now viewed as an organ that can be targeted to exert anti-diabetic effects associated with improvements of dyslipidemia and insulin secretion as well as decrease insulin resistance in T2D^20^,^21^,^22^. Finally, the central nervous system controls thermogenesis via sympathetic nervous system^23^,^24^. More precisely, hypothalamic proopiomelanocortin (POMC) neurons are now considered as major type of neurons implicated in the control of energy expenditure^25^,^26^.

Thus, the present work aims to: 1) determine whether chronic icv apelin infusion in mice induces hypothalamic and/or systemic inflammation; and 2) investigate the peripheral consequences of this chronic treatment on whole-body energy expenditure, which is profoundly altered during insulin resistance^4^,^27^; and on thermogenesis BAT function which is under the control of hypothalamus in normal and pathological conditions^28^,^29^.

## Results

### Chronic icv apelin increases the expression of hypothalamic pro-inflammatory markers

We have previously shown that over-expression of apelin in the hypothalamus *via* lentiviral approaches provokes a T2D phenotype in normal mice by increasing hepatic gluconeogenesis and glycogenolysis^12^,^30^. To further explore the impact of chronic exposure of apelin in the brain, we developed a mice model chronically perfused with apelin in lateral ventricle. We measured the expression and activity of a hepatic enzyme implicated in gluconeogenesis and glycogenolysis. In accordance with our previous study^12^, we demonstrated an increase in *Hepatic glucose 6-phosphatase (G6Pase)* mRNA expression (Suppl. Fig. 1a) and activity (Suppl. Fig. 1a) in icv apelin treated mice that could contribute to the fasting hyperglycemia and therefore the T2D phenotype observed in this experimental model^31^. Again, we found that chronic apelin is associated with an increase in plasma noradrenalin (Suppl. Fig. 1b), but not adrenalin (Suppl. Fig. 1b), confirming the over-activation of sympathetic tone as previously described^12^. Thus we conclude that the present icv model is comparable to our previous model of chronic expression of apelin.

**Figure 1:**
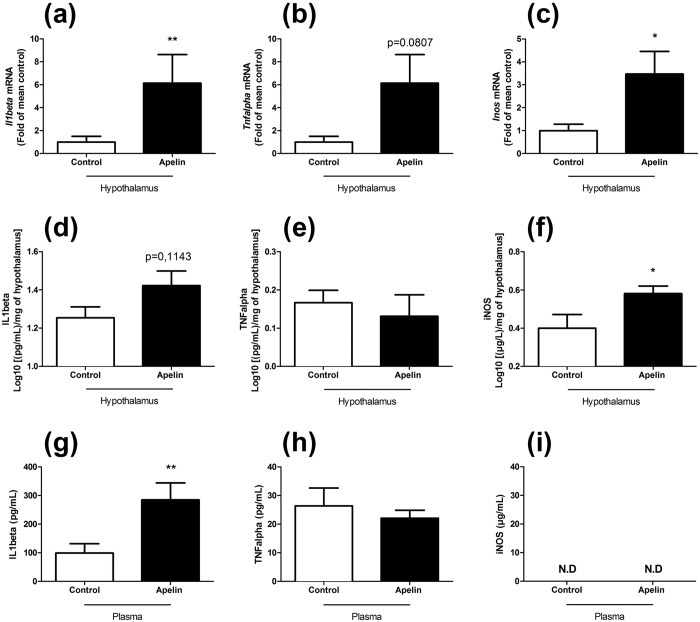
Chronic icv apelin increases the expression of hypothalamic and plasma pro-inflammatory markers. Effect of chronic apelin treatment (Apelin) versus chronic aCSF treatment (Control) on hypothalamic (**a**) *Il1beta* (**b**) *Tnfalpha* and on (**c**) *Inos* mRNA expression. Effect of chronic apelin treatment (Apelin) versus chronic aCSF treatment (Control) on IL1beta (**d**), TNFalpha (**e**) and iNOS (**f**) hypothalamic levels. Effect of chronic apelin treatment (Apelin) versus chronic aCSF treatment (Control) on IL1beta (**g**), TNFalpha (**h**) and iNOS (**i**) plasma levels. Experiments were performed with a set of 5–7 9 mice in each group. *p < 0.05, **p < 0.01. N.D: Not Determined.

Given that, first, a positive correlation exists between apelin and inflammation during metabolic diseases^32^ and, second, an inflammatory induction was demonstrated in microglial cells culture in response to apelin^33^, we have decide to measure the variations of specific pro-inflammatory markers expressed in the entire hypothalamus. We found that chronic icv treatment significantly increases *Interleukin-1 beta (Il1beta)* (Fig. 1a) *and Inducible Nitric Oxide Synthase (Inos)* (Fig. 1c) mRNA expression, while a trend is noted for *Tumor Necrosis Factor alpha (Tnfalpha)* (p = 0.0807, Fig. 1b), thereby suggesting an increased inflammation in this model. Changes at the mRNA levels were associated with an increase in hypothalamic iNOS protein expression, but not for IL1beta and TNFalpha (Fig. 1d–f). These effects are specific to the hypothalamus since no significant variations are observed for the three markers in the brain stem (Suppl. Fig. 2a–c) or cortex (Suppl. Fig. 2d–f). Moreover, this hypothalamic inflammation is associated with an increase in plasma IL1beta (Fig. 1g) but not TNFalpha (Fig. 1h). The plasma iNOS expression was not detected in our samples (Fig. 1i).

### Chronic icv apelin does not modify body weight and food intake, but stimulates hypothalamic POMC neurons

**Figure 2:**
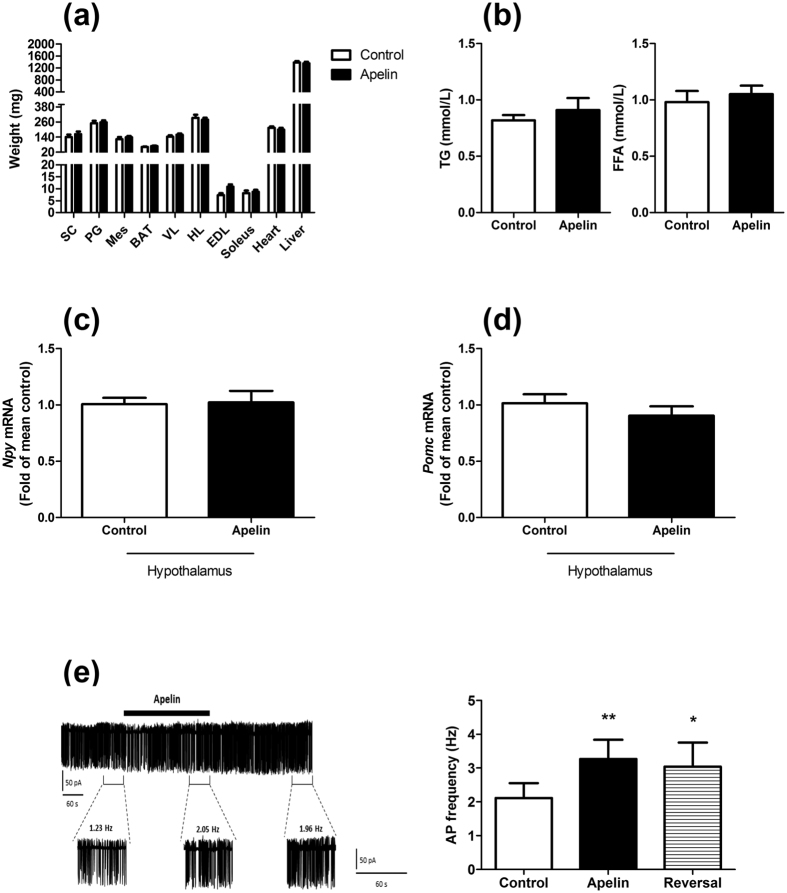
Apelin depolarizes POMC neurons in the hypothalamus without effect on food intake and tissue weight. Effect of chronic apelin treatment (Apelin) versus chronic aCSF treatment (Control) (**a**) on tissues weight (SC: subcutaneous adipose tissue, PG: perigonadal adipose tissue, Mes: mesenteric adipose tissue, BAT: brown adipose tissue, VL: vastus lateralis (muscle); HL: muscle; EDL: extensor digitorum longus (muscle)); (**b**) on triglycerides (TG) and free fatty acids (FFA) plasma levels; (**c**) on hypothalamic *Npy* mRNA expression and; (**d**) on hypothalamic *Pomc* mRNA expression. (**e**) (Left panel) Representative cell-attached recording of a POMC-GFP neuron activated by apelin (thick black bar under the trace). Panels below the trace represent enlarged 60 seconds recording periods with average action potential frequency before, during and after apelin bath application. (Right panel) Quantification of action potential (AP) frequency of POMC before (control; over the last 60 s before apelin application), during (apelin, over the last 60 seconds of apelin application) and after (reversal, over 60 seconds, 5 minutes after apelin application) apelin application. Experiments were performed with a set of 6–9 mice in each group. **p < 0.01; *p < 0.05 vs. control.

We have previously found that chronic perfusion of icv apelin did not modify body weight and food intake^31^. Here we show that tissue weights are not modified by icv apelin treatment (Fig. 2a) thereby supporting the absence of body weight gain. Plasma triglycerides (TG) and free fatty acids (FFA) are not affected (Fig. 2b). The absence of effect on food intake is correlated with the lack of variations of neuropeptide Y (NPY) mRNA and proopiomelanocortin (POMC) expressions in the hypothalamus between control and apelin treated mice (Fig. 2c,d). As APJ receptor is strongly expressed on POMC neurons compared to NPY neurons^18^, we have measured the impact of apelin on these neurons by electrophysiological recordings in brain slices from POMC-eGFP mice. Apelin (20 nM) increases action potential frequency in ~50% of POMC-expressing neurons (n = 9/19) by 57.9 ± 11.3% (control: 2.11 ± 0.44 vs. apelin: 3.26 ± 0.57 Hz; n = 9; p < 0.05) (Fig. 2e). In general, POMC neurons do not return to basal firing rates until at least 10 minutes after peptide application. This sustained effect is consistent with the effect of apelin observed on vasopressin neurons^23^.

### Chronic icv apelin decreases energy expenditure, without modification of ambulatory activity

**Figure 3:**
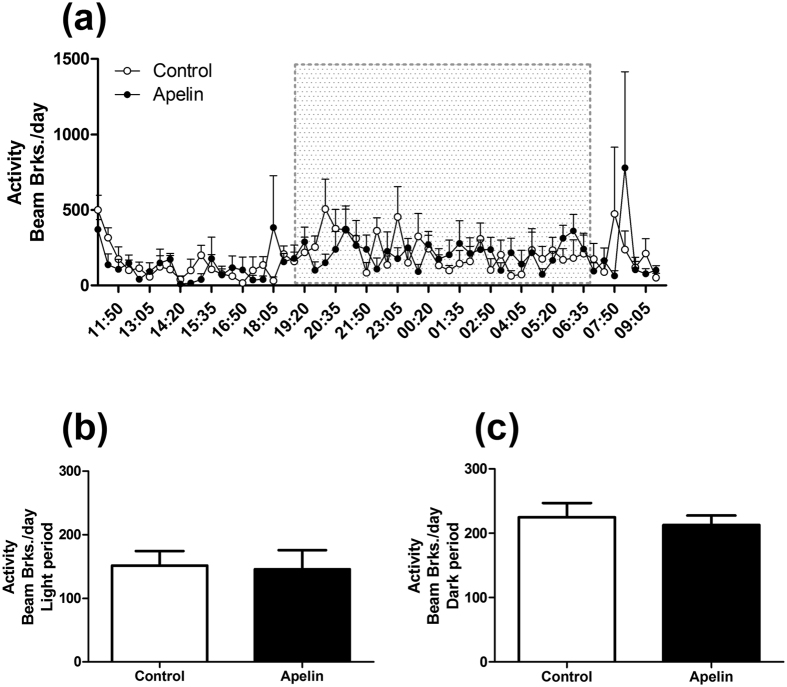
Chronic icv apelin does not modulate ambulatory activity. Effect of chronic apelin treatment (Apelin) versus chronic aCSF treatment (Control) (**a**) on global ambulatory activity; (**b**) on ambulatory activity during the light period; (**c**) on ambulatory activity during the dark period. Experiments were performed with a set of 5–6 mice in each group.

**Figure 4:**
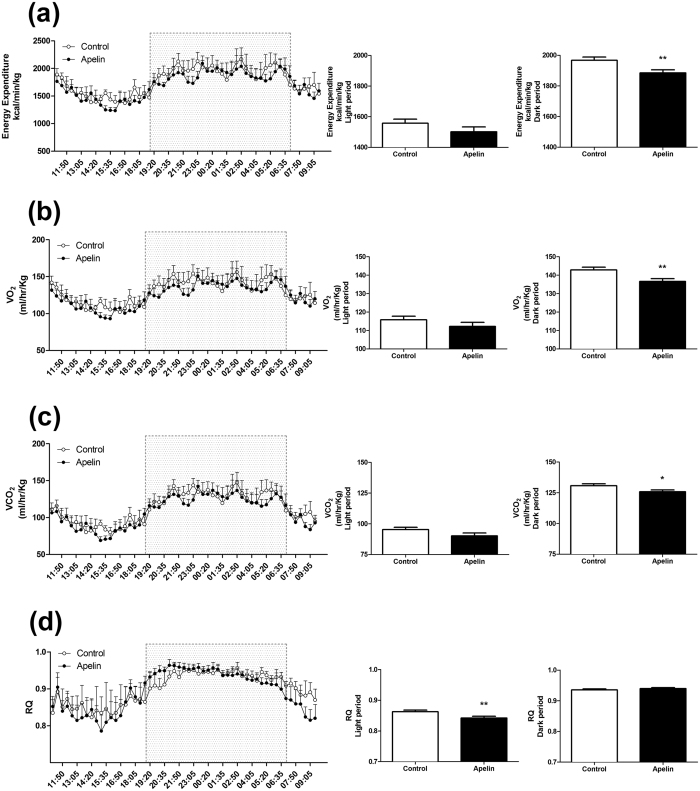
Chronic icv apelin decreases energy expenditure. Effect of chronic apelin treatment (Apelin) versus chronic aCSF treatment (Control) (**a**) on energy expenditure with a focus during dark and light period; (**b**) on VO_2_ with a focus during dark and light period; (**c**) on VCO_2_ with a focus during dark and light period and; (**d**) on Respiratory Quotient (RQ) with a focus during dark and light period. Experiments were performed with a set of 5–6 mice in each group. *p < 0.05, **p < 0.01.

We previously demonstrated that hypothalamic apelin was involved in the onset of insulin resistance and T2D^31^, which are known to be associated with altered energy expenditure^4^. To explore this hypothesis, we measured the effects of chronic icv apelin on energy expenditure by indirect calorimetry. Apelin treatment do not modify the ambulatory activity during the dark and light periods (Fig. 3a–c). Apelin-treated mice showed decreased energy expenditure, oxygen consumption (VO_2_) and carbon dioxide production (VCO_2_) (Fig. 4a–c) during the dark period (i.e., during the feeding period of the mice)^31^. This effect is associated with a slight but significantly reduced respiratory quotient during the light phase (Fig. 4d).

### Chronic icv apelin alters thermogenesis and BAT function

BAT contains a high density of mitochondria with high amounts of UCP1 allowing the uncoupling of fatty acid oxidation from ATP production to generate heat^34^.

**Figure 5:**
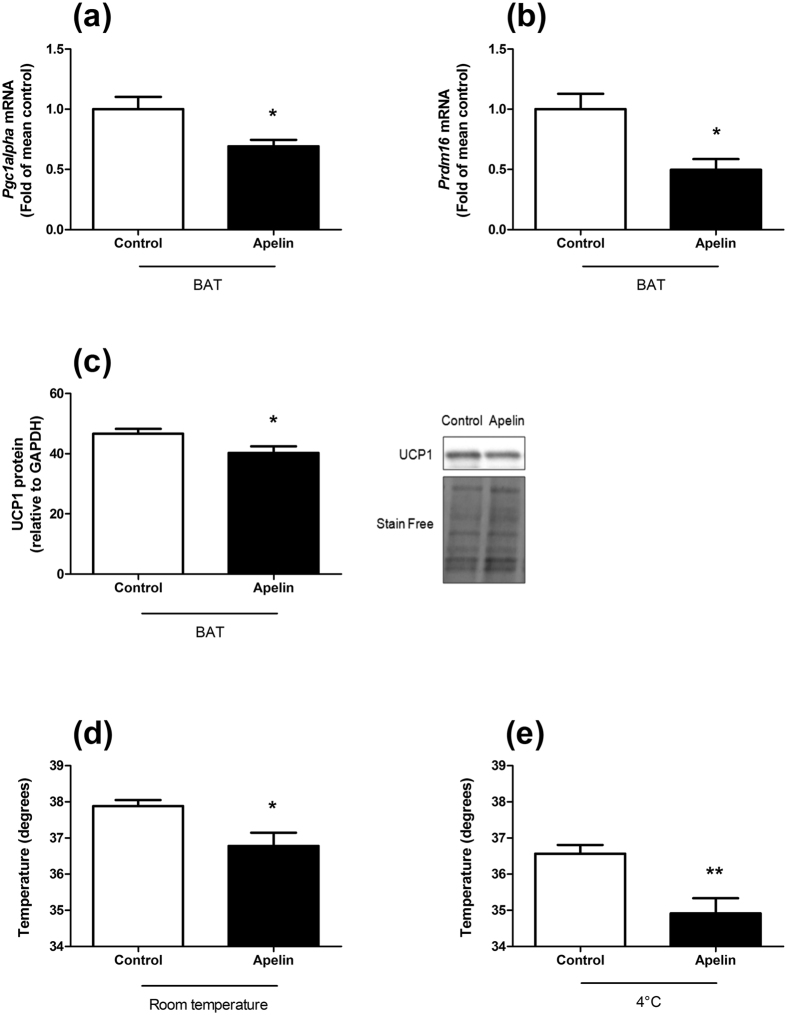
Chronic icv apelin mice present an alteration of thermogenesis. Effects of chronic apelin treatment (Apelin) versus chronic aCSF treatment (Control) (**a**) on BAT *Pgc1alpha* mRNA expression; (**b**) on BAT *Prdm16* mRNA expression; (**c**) on BAT UCP1 protein level; (**d**) on body temperature at room temperature and; (**e**) on body temperature at 4 °C. Experiments were performed with a set of 4–7 mice in each group. *p < 0.05, **p < 0.01.

We found that chronic icv apelin perfusion decreased the expression of *Peroxisome proliferator-activated receptor 1 alpha (Pgc1alpha)* mRNA in BAT (Fig. 5a) which is a key regulator of mitochondrial biogenesis^35^, and *PR domain containing 16 (Prdm16)* mRNA (Fig. 5b), a key transcriptional cofactor involved in BAT differentiation^36^. This result suggests that chronic icv apelin perfusion decreases mitochondrial biogenesis and reduces BAT activity. In addition, UCP1 protein level is significantly decreased in BAT in response to chronic icv apelin perfusion (Fig. 5c), validating a disruption of its function. This was associated with a decrease in body temperature at room temperature (Fig. 5d) and an altered response to cold exposure (Fig. 5e) in mice perfused with chronic icv apelin, thereby contributing to explain the decreased energy expenditure upon apelin treatment. In the BAT, genes encoding lipids or glucose transports markers are not modified between the 2 groups of mice (Suppl. Fig. 3a–f).

## Discussion

In the present study, we have demonstrated that central chronic perfusion of apelin, at a dose known to induce insulin resistance^31^, increases hypothalamic and plasma inflammation, decreases energy expenditure and thermogenesis associated with an alteration of BAT function.

The role of apelin as anti-inflammatory marker in peripheral tissues is still a matter of debate. For example, data from literature suggest that apelin 1) has an anti-inflammatory role in heart^37^, 2) prevents UVB-induced inflammation^38^ or 3) inhibits macrophages inflammation in aorta^39^. Conversely, we have previously found a positive correlation between inflammation and apelin/APJ expression in intestinal and adipose tissue of obese/diabetic mice^40^. In the brain, we have demonstrated that high levels of apelin (mimicking the quantity of apelin present in the hypothalamus of obese/diabetic mice)^18^, generate hypothalamic inflammatory state with the induction of *Il1beta* and *Tnfalpha* expression^12^. Along the same line, Chen *et al*. have recently described that apelin induces inflammatory signalling pathways in the BV2 microglial cell line^33^. In the present study, brain *Il1beta* and *Inos* mRNA levels were increased in apelin-treated mice, reinforcing the inflammatory role of apelin in the hypothalamus. In parallel, iNOS protein expression, but not IL1beta, is significantly increased in the hypothalamus of apelin-treated mice. These data are in accordance with the variation of mRNA expression except for IL1b. This last result could be justify by the existence of a diurnal rhythm of IL1b expression in specific brain regions including the hypothalamus^41^. In addition, microglial inflammatory responses are controlled by an intrinsic circadian clock including TNFa, IL1b and IL6^42^, thereby suggesting that time is a factor to consider when investigating inflammatory interventions on the brain^42^. Whether this phenomenon occur in the present experimental setting remain to be investigated.

We have previously demonstrated that chronic icv apelin infusion triggered the onset of glucose homeostasis disorders, e.g., glucose intolerance and insulin resistance^31^. However, altered regulation of energy expenditure is among the key features associated with obesity and T2D. A plethora of targets have been identified as major regulators of energy balance, playing roles in energy intake and/or expenditure^43^,^44^. Here, we have discovered that apelin chronically perfused in the brain reduced energy expenditure during the feeding period. Interestingly, apelin and its receptor are expressed in hypothalamic POMC neurons and control the release of alpha-melanocyte-stimulating hormone (alphaMSH)^18^. Our data, combined with the literature, strongly suggest that apelin controls energy homeostasis by different mechanisms. Therefore, we speculate that the altered energy expenditure observed during obesity might occur *via* apelin-dependent mechanisms on POMC neurons since we demonstrate that apelin activates these neurons in the arcuate nucleus. The absence of effect on food intake despite the modification of POMC activity in response to apelin fits with the demonstration that hypothalamic POMC neurons present a functional heterogeneity^45^. In fact, data from literature support the hypothesis that distinct populations of POMC neurons are implicated in the control of food intake and energy metabolism^45^. Activation of POMC neurons is usually associated with an increase in energy expenditure. Conversely, our data show a decrease in energy expenditure linked to a POMC neurons depolarization. In the hypothalamus, POMC neurons are considered as target of apelin actions. Apelin is also expressed on POMC neurons, and quantitative analysis demonstrated that 89% of apelin neurons contained POMC, whereas less than 10% contained NPY. However, numerous NPY nerve fibers are detected close to apelin neuronal cell bodies implying potential control of NPY on POMC/Apelin neurons activity^13^. We have shown that apelin released from POMC neurons may stimulate α-MSH release in an autocrine manner^18^. Similar effect could be proposed on NPY neurons. Another explanation may be that apelin acts on glutamatergic neurons located on paraventricular nucleus of the hypothalamus. Thus, stimulation of these neurons by apelin generates an activation of the sympathetic nervous system^46^ which could explain the phenotype of chronic icv apelin treated mice. In fact, the role of hypothalamic apelin in the control of glucose metabolism remains to be unraveled, and is complex. Again, data from the literature show that stimulation of MC4R in the brain (i.e., the α-MSH receptor) stimulates the activation of sympathetic nervous system^47^. This is similar to that we have observed in our experimental model^12^ thereby explaining the onset of the diabetic phenotype (i.e., hyperglycemia and decrease of energy expenditure) in chronic icv apelin perfused mice. To conclude, we cannot rule out the possible presence of APJ receptor on specific subpopulations of POMC neurons implicated in the control of glucose metabolism. Future experiments will be performed to unravel this complex role of central apelin on energy homeostasis.

We do not observe significant changes in body weight and fat mass development after two-weeks of apelin infusion into the brain, whereas energy expenditure was significantly decreased. We cannot exclude that a longer period of treatment could significantly affect body weight and fat storage, we have found no significant variation of body weight after one month of icv apelin perfusion (personal communication). Another potential speculation is that chronic apelin treatment slightly but significantly reduced the respiratory quotient during the light phase. This finding might support a trend to shift towards lipid oxidation instead of carbohydrate utilization as previously described with peripheral long term apelin treatment^48^. Thus, this may contribute to the lack of increase in body weight and fat deposition during the treatment period, compensating the reduced energy expenditure at the onset of the pathology. In fact, we have previously published that chronic icv apelin increases hepatic neoglucogenesis^12^ hence contributing to the development of the diabetic phenotype in mice. Recently, Perry *et al*.^49^ have demonstrated that the increase in hepatic neoglucogenesis observed in obese/diabetic mice is due to an elevation in hepatic acetyl coA which results from the increased rates of white adipose tissue lipolysis (i.e., oxidation). Taken together, our data support the hypothesis that, in spite of a decrease in energy expenditure associated with BAT dysfunction, the increase in hepatic neoglucogenesis observed in our experimental model favours the slight increase in lipid oxidation.

T2D is associated with a decrease in energy expenditure and alteration of metabolic tissues functions, leading to an altered nutrients utilisation and insulin resistance. We have previously described that acutely mimicking the levels of apelin measured in the brain of obese and diabetic mice increase hepatic glucose production during the fasted period, a feature of T2D^12^. In this study, we found an increase in *G6Pase* mRNA expression and enzymatic activity, confirming power full brain to liver axis, and that the liver is a target of high levels of apelin. In addition to the liver, we demonstrated in this study that BAT function and thermogenesis were also modified in response to chronic icv apelin. We found that the pre-diabetic phenotype induced by icv apelin treatment was associated with a decrease in *Pgc1alpha* and *Prdm16* mRNA expression and a decrease in UCP1 proteins level in BAT. These data confirm the deleterious effect of chronic central apelin injection on energy metabolism^12^,^13^ as opposed to its peripheral beneficial effects on insulin sensitivity^11^. Here, we discovered that central apelin exerts inverse effect on BAT function as opposed to its direct effects on the brown adipogenic capacity and browning^28^. This last result fits with the concept that we have previously highlighted demonstrating that over-expression of apelin in the brain participates to the establishment of T2D^30^,^50^,^51^. Therefore, we speculate that the decrease in energy expenditure observed in these mice might be explained by the reduced expression of key factors characterizing BAT activity. Indeed, UCP1 is generally recognized as the defining marker of brown fat and interacts strongly with PGC1alpha, the master regulator of UCP1-mediated thermogenesis in BAT^52^,^53^. Moreover, numerous data from the literature have demonstrated that PGC1alpha acts as a crucial factor in the control of mitochondrial biogenesis and energy status^54^. Thus, it has been considered as a potential therapeutic target for treating mitochondrial dysfunction associated with insulin resistance^55^. Although Pgc1a and Prdm16 mRNA expression have been previously associated with mitochondrial biogenesis (i.e., real quantification), our data strongly suggest this association but does not show this link in our set of data. In addition, PRDM16 was considered as an activator of brown fat cell identity, at least in part by stimulating PGC1alpha expression and activity^36^. Taken together, these molecular modifications involved in the key thermogenic role of BAT are strongly linked to the decrease in rectal temperature, which mirror basal metabolic rate, but also with the lower energy expenditure observed in response to chronic icv apelin in mice. Here, we discovered that central apelin significantly increases the level of plasma cytokine IL1beta. Recently, Goto *et al*. have demonstrated that IL1beta suppresses cold-induced thermogenesis in adipocytes^56^ which brings a potential molecular explanation to decipher the metabolic phenotype of our central apelin-treated mice. Furthermore, a possible link between plasma IL1beta and thermogenesis could be UCP1 since IL1beta is able to decrease UCP1 expression in adipocytes^56^ similar to that observed in our study.

In conclusion, the central regulation of energy metabolism has become a hot topic in recent years, and it was demonstrated that the hypothalamic structures are largely implicated. Taken together, we found that increased brain apelin levels, induce a local inflammatory state in the hypothalamus, but is also correlated with an increase in plasma IL1beta levels. This is associated with a reduced energy expenditure likely paralleled by a reduced BAT activity and thermogenesis. Thus, data from the literature and from the present study suggest that hypothalamic inflammation generates a pre-T2D state *via* multiple peripheral organ dysfunctions, including BAT.

## Materials and Methods

### Animals

This study was performed in accordance with appropriate guidelines from the Guide for the Care and Use of Laboratory Animals of the European Council. All experimental protocols were approved by the Animal Care and Ethics Committee of US006/CREFE (CEEA-122) with permit N° C3155508. C57Bl6/J mice were obtained from Charles River Laboratory (l’Arbresle, France). The mice were housed in Specific Pathogen Free zone in a constant temperature (20–22 °C) and humidity (50–60%) animal room with a 12/12 h light/dark cycle (lights off at 7:00 am) and with free access to food and water over the 24 h period. All injections and experiments were performed in 13- to 15-week-old males. Different sets of mice were used for the experiments described below.

### Surgical procedures

For *in vivo* chronic infusions, an indwelling icv catheter (Alzet Brain Perfusion Kit 3, 1–3 mm, Charles River, L’Arbresle, France, −1 mm lateral, −0.2 mm anteroposterior from the bregma and −1.7 mm deep) was implanted in anesthetised mice with isoflurane (Abbott, Rungis, France). The icv catheter was connected to an osmotic mini-pump (Model 2004, Alzet; Cupertino, CA) as previously described^4^,^31^.

### Chronic infusion of apelin

Briefly, an osmotic mini-pump system connected to the lateral ventricle delivered either aCSF or apelin-13 (20 nM) over 2 weeks, at a rate of 0.25 μl/h as previously used^31^. At this dose and in this experimental model, icv apelin provoked fasting hyperglycaemia and hyperinsulinaemia, glucose intolerance, insulin intolerance and insulin resistance^31^. It is worth noting that the stability of apelin and the duration of the treatment were compatible with the stability of the molecule in such experimental conditions, as previously described by several groups^31^,^46^. Moreover, we tested the apelin from the pumps after the 2 weeks of treatment, and was still active. To demonstrate this, apelin from the pumps harvested at the end of the experiments was directly injected icv in another set of mice. Here, we found that acute icv apelin has conserved its central action and generated a fasting hyperglycemia (data not shown) as previously described^31^.

The mice were housed individually during the 2 weeks and the temperature was taken in the end of this chronic infusion.

## Dissection

The mice were fasted for 6 h and sacrificed. BAT, hypothalamus and liver were removed, frozen and powdered in liquid nitrogen and were kept at −80 °C until the experiments. Plasmas were also taken from mice for ELISA assays.

### Plasma assays

Plasma IL1beta (Sigma Aldrich, MO, USA), TNFalpha (Invitrogen, MD, USA) and iNOS (LSBio, WA, USA) levels were measured with ELISA kits using manufacturer’s instructions. Catecholamine content of the plasma was determined as previously described in details using high-performance liquid chromatography coupled with electrochemical detection after alumina extraction^12^. Plasma TG and FFA were measured as previously described^57^.

### RT-qPCR

Total RNA from tissues were prepared using the TriPure reagent (Roche, Basel, Switzerland) as previously described^57^,^58^. Primer sequences for the targeted mouse genes are presented in Table 1. cDNA were prepared by reverse transcription of 1 μg of total RNA, using a Reverse Transcription System kit (Promega, Leiden, the Netherlands). Real-time PCR was performed with the StepOnePlus^TM^ real-time PCR system and software (Applied Biosystems, Den Ijssel, the Netherlands) using Mesa Fast qPCR^TM^ (Eurogentec, Seraing, Belgium) for detection, according to the manufacturers’ instructions. *Beta2microglobulin* was chosen as housekeeping gene in the liver and BAT; *Rpl19* was chosen as housekeeping gene in the brain since *Rpl19* expression did not varied during rodent brain development^59^. All of the tissues were run in duplicate on a single 96-well reaction plate (MicroAmp Optical, Applied Biosystems), and data were analysed according to the 2^−ΔΔCT^ method. The identity and purity of the amplified product were assessed by analysis of the melting curve, performed at the end of amplification. Primer sequences for the targeted mouse genes are presented on Table 1.

### Enzymatic activity

Hepatic glucose-6-phosphatase activity was determined as previously described in details^12^.

### Indirect calorimetric and ambulatory activity

Twenty-four-hour energy expenditure was measured by indirect calorimetry (Oxylet; Panlab Bioseb, Chaville, France) during week 2 of the treatment, as previously described^4^. Oxygen consumption (VO_2_), carbon dioxide production (VCO_2_), Respiratory quotient (RQ) (calculated according to the following formula: VCO_2_/VO_2_), energy expenditure (calculated according to the following formula: 1.44xVO_2_x[3.815 + 1.232 x RQ], and ambulatory activity were measured in control and apelin-treated mice. Oxygen consumption and carbon dioxide production were measured over a 24-h period. The ambulatory activities of the mice were monitored using an infrared photocell beam interruption method (Sedacom; Panlab-Bioseb). At the end of the protocol, the mice were sacrificed, and peripheral tissues were weighed.

### Body temperature

Body temperature was measured at week 2 during the fed period using a thermometer for small animals (Thermometer TK 98802; Bioseb) and after one hour at 4 °C, as previously described^4^.

### Western Blot

BAT tissues were homogenized in a buffer containing 50 mmol/L HEPES, pH 7.4, 2 mmol/L EDTA, 150 mmol/L NaCl, 30 mmol/L NaPO4, 10 mmol/L NaF, 1% Triton X-100, 10 mL/mL protease inhibitor (Sigma-Aldrich), 10 mL/mL phosphatase I inhibitor (Sigma-Aldrich), 10 mL/mL phosphatase II inhibitor (Sigma-Aldrich), and 1.5 mg/mL benzamidine HCl. Tissue homogenates were centrifuged for 25 min at 15.000 g, and supernatants were stored at −80 °C. Solubilized proteins from muscle tissue were run on a 4–20% Criterion SFX Tris-HCl (Bio-Rad, Hercules, CA), transferred onto nitrocellulose membrane (Hybond ECL, GE Healthcare, Buckingamshire, UK), and incubated with the primary antibodies for UCP1 (Abcam #10983; 1/3000). Subsequently, immunoreactive proteins were determined by enhanced chemiluminescence reagent (Clarity Western ECL (Biorad) and visualized by exposure to CCD Imaging Chemidoc MP (Biorad, Hercules, CA). Volume density was analyzed for each band and normalized to total transferred proteins determined by stain free.

### Electrophysiology

Brain slices (250 μm) were prepared from adult POMC-eGFP mice (6–8 weeks old; POMC-EGFP1Low/J, The Jackson Laboratory) as previously described^60^. Slices were incubated at room temperature, in oxygenated extracellular medium containing (in mM): 118 NaCl, 3 KCl, 1 MgCl_2_, 25 NaHCO_3_, 1.2 NaH_2_PO_4_, 1.5 CaCl_2_, 5 Hepes, 2.5 D-glucose (osmolarity adjusted to 310 mOsM with sucrose, pH 7.3) for a recovery period (at least 60 minutes). Once in the recording chamber, slices were perfused at 2–3 ml/min with the same extracellular medium. Slices were observed with a Nikon microscope (EF600) outfitted for fluorescence (fluorescein filter) and IR-DIC videomicroscopy. Viable arcuate nucleus POMC neurons were visualized using a X40 water immersion objective (Nikon) with a fluorescence video camera (Nikon). Borosilicate pipettes (4–6 MΩ; 1.5 mm OD, Sutter Instrument) were filled with filtered extracellular medium. Cell-attached recordings were performed using a Multiclamp 700B amplifier, digitized using the Digidata 1440A interface and acquired at 3 kHz using pClamp 10.3 software (Axon Instruments). Pipettes and cell capacitances were fully compensated. After a stable baseline was established, apelin (20 nM of [Pyr]apelin-13, Bachem) was perfused for 4–5 minutes. This dose of apelin (20 nM) corresponds to the quantity of apelin found in a half hypothalamus of mice^31^. Addition of this dose of apelin could increase the level of apelin in the hypothalamus by about 1.5 fold, thereby mimiking the quantity of hypothalamic apelin observed in obese/diabetic mice^31^. The firing activity was measured over the last minute of the apelin perfusion, 10 minutes after the perfusion and compared with the firing rate measured 1 minute before the perfusion.

### Statistical analysis

The data are expressed as the means ± SEMs. Differences between the two groups were assessed by Student’s unpaired *t* test or one way ANOVA, followed by post-hoc Bonferroni-test, as appropriate. The data were analysed using GraphPad Prism software, version 5.00 for Windows (GraphPad Software, San Diego, CA, USA). The results were considered statistically significant at p < 0.05.

## Additional Information

**How to cite this article**: Drougard, A. *et al*. Central chronic apelin infusion decreases energy expenditure and thermogenesis in mice. *Sci. Rep.* **6**, 31849; doi: 10.1038/srep31849 (2016).

## Supplementary Material

Supplementary Information

## Figures and Tables

**Table 1:** Sequence of the oligonucleotide primer sets used in RT-PCR analysis.

Target genes	Primer sequence 5′ to 3′	
	Sense	Antisense
Beta2microglobulin	CACTGACCGGCCTGTATGC	GGGTGGCGTGAGTATACTTGAATT
Cd36	GGACATACTTAGATGTGGAACCCATA	TGTTGACCTGCAGTCGTTTTG
Fabp4	TGTGGGAACCTGGAAGCTTGTC	TCTGACCGGATGGTGACCAAA
Fatp1	GACAAGCTGGATCAGGCAAGC	AGTGAGGCCACAGAGGCTGTT
Fiaf	CAATGCCAAATTGCTCCAATT	TGGCCGTGGGCTCAGT
Glut1	GGTGTGCAGCAGCCTGTGT	CACAGTGAAGGCCGTGTTGA
Glut4	CCGGATTCCATCCCACAAG	CATGCCACCCACAGAGAAGA
G6pase	ACGTATGGATTCCGGTGTTTG	CAGCTGCACAGCCCAGAA
Inos	CACCTTGGACTTCACCCAGT	ACCACTCGTACTTGGGATGT
Il1beta	CAACCAACAAGTGATATTCTCGATG	GATCCACACTCTCCAGCTGCA
Npy	CAGAAAACGCCCCCAGAAC	CGGGAGAACAAGTTTCATTTCC
Pgc1alpha	AAAGGATGCGCTCTCGTTCA	GGAATATGGTGATCGGGAACA
Pomc	AGGCCTGACACGTGGAAGAT	AGCAGGAGGGCCAGCAA
Prdm16	CAGCACGGTGAAGCCATTC	GCGTGCATCCGCTTGTG
Rpl19	GAAGGTCAAAGGGAATGTGTTCA	CCTTGTCTGCCTTCAGCTTGT
Tnfalpha	TGGGACAGTGACCTGGACTGT	TTCGGAAAGCCCATTTGAGT

CD36 (cluster of differenciation 36); Fabp4 (fatty acid-binding protein 4); Fatp1 (fatty acid transport protein 1); Fiaf (fasting-induced adipose factor); Glut1 (glucose transporter 1); Glut4 (glucose transporter 4); G6pase (glucose-6-phosphatase); Inos (inducible nitric oxide synthase); Il1beta (interleukin 1 beta); Npy (neuropeptide Y); Pgc1alpha (peroxisome proliferator-activated receptor 1 alpha); Pomc (pro-opiomelanocortin); Prdm16 (PR domain containing 16); Rpl19 (60S ribosomal protein L19); Tnfalpha (tumour necrosis factor alpha).
