# Supplementary material for: Central chronic apelin infusion decreases energy expenditure and thermogenesis in mice
Source: Sci Rep. 2016 Aug 23;6:31849. doi: 10.1038/srep31849 (PMC4994119; doi:10.1038/srep31849)
Supplement: Supplementary Information [file srep31849-s1.doc]

**Supplementary figures and legends**

Central chronic apelin infusion decreases energy expenditure and thermogenesis in mice

**Anne Drougard ^1,2,*,§^, Audren Fournel ^1,2,*^, Alysson Marlin ^1,2^, Etienne Meunier ^3^, Thibaut Duparc ^1,2^, Katie Louche ^1^, Aurelie Batut ^1^, Alexandre Lucas ^1^, Sophie Le-Gonidec ^1^, Jean Lesage ^5^, Xavier Fioramonti ^6^, Cedric Moro ^1^, Philippe Valet ^1,2^, Patrice D. Cani ^2,4*^, Claude Knauf ^1,2*^**

*^1^ Institut National de la Santé et de la Recherche Médicale (INSERM), U1048, Université Paul Sabatier, UPS, Institut des Maladies Métaboliques et Cardiovasculaires (I2MC), CHU Rangueil, 1 Avenue Jean Poulhès, BP84225, 31432 Toulouse Cedex 4, France*

*^2^ NeuroMicrobiota, European Associated Laboratory (EAL) INSERM/UCL*

*^3^ Focal Area Infection Biology, Biozentrum, University of Basel, Klingelbergstrasse 50/70 CH-4056 Basel, Switzerland*

*^4^ Université Catholique de Louvain (UCL), Louvain Drug Research Institute, LDRI, Metabolism and Nutrition research group, WELBIO, WELBIO (Walloon Excellence in Life sciences and BIOtechnology), Av. E. Mounier, 73 B1.73.11, B-1200, Brussels, Belgium*

*NeuroMicrobiota, European Associated Laboratory (EAL) INSERM/UCL*

*^5^ Université de Lille, Unité environnement périnatal et santé, EA 4489, Équipe malnutrition*

*maternelle et programmation des maladies métaboliques, Université de Lille1, Bâtiment SN4,*

*59655 Villeneuve d’Ascq, France*

*^6^ Centre des Sciences du Goût et de l'Alimentation, CNRS, INRA, Univ. Bourgogne Franche-Comté, F-21000 Dijon, France*

§ present address : Max Planck Institute of Immunobiology and Epigenetics, Stübeweg 51, 79108 Freiburg, Germany.


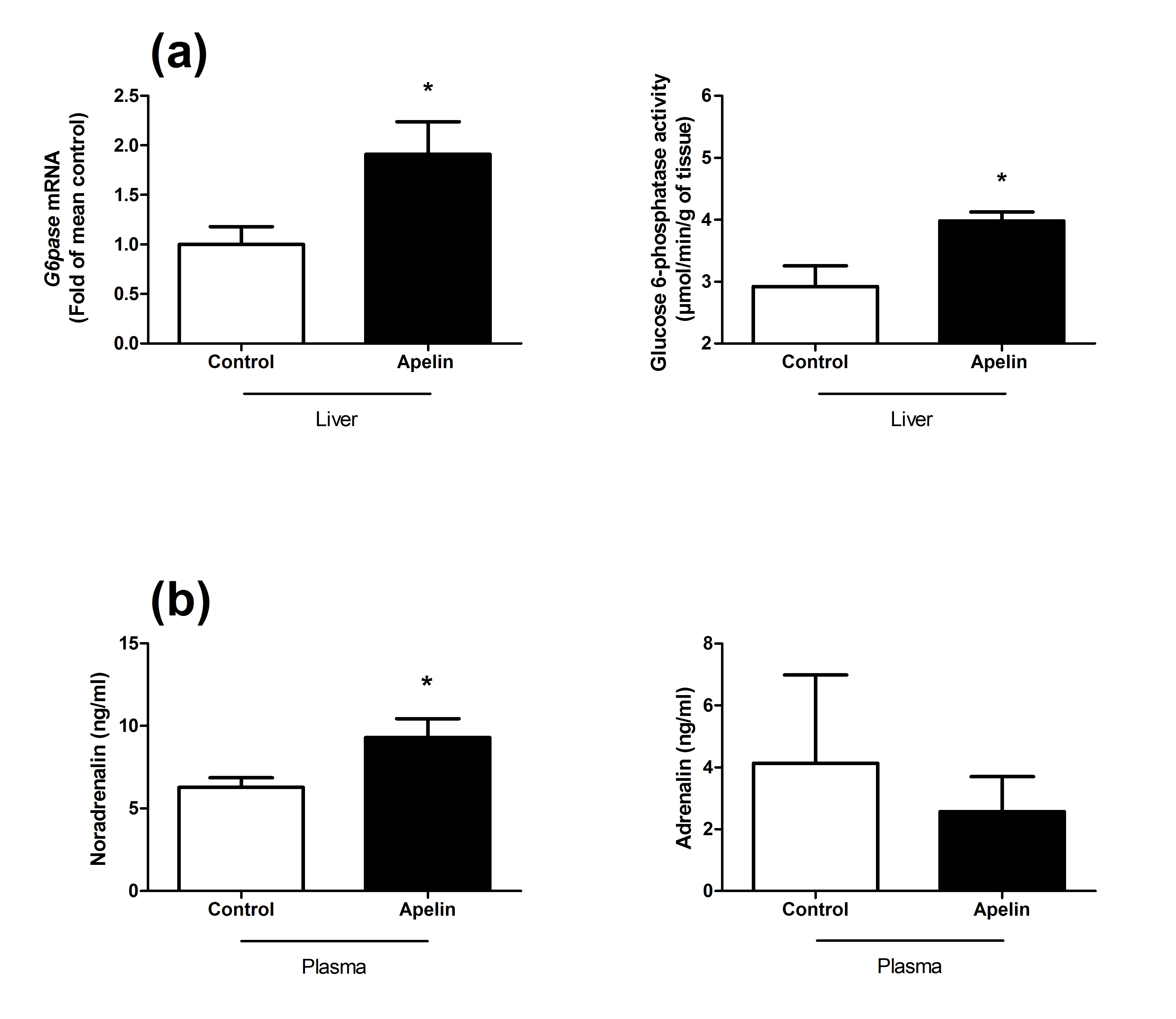


**Supplementary Figure 1.** Effects of chronic apelin treatment (Apelin) versus chronic aCSF treatment (Control) (a) on liver *G6pase* mRNA expression and Glucose 6-phosphatase activity and; on (b) Noradrenalin and Adrenalin plasma levels. Experiments were performed with a set of 4-9 mice in each group. *p<0.05.


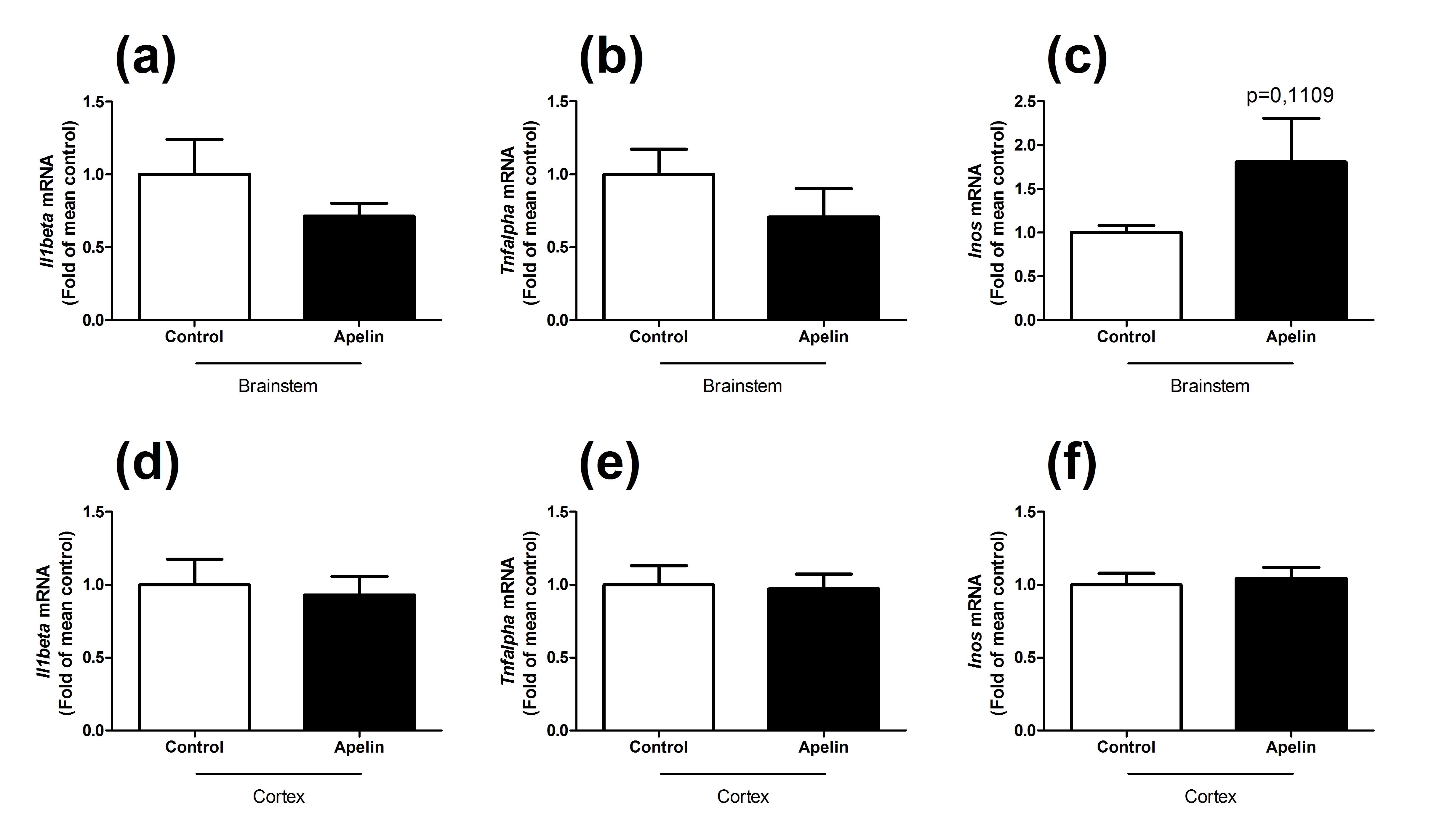


**Supplementary Figure 2.** Effect of chronic apelin treatment (Apelin) versus chronic aCSF treatment (Control) on brainstem (a) and cortex (d) *Il1beta* mRNA expression; on brainstem (b) and cortex (e) *Tnfalpha* mRNA expression; and on (c) and cortex (f) *Inos* mRNA expression. Experiments were performed with a set of 4-6 mice in each group.


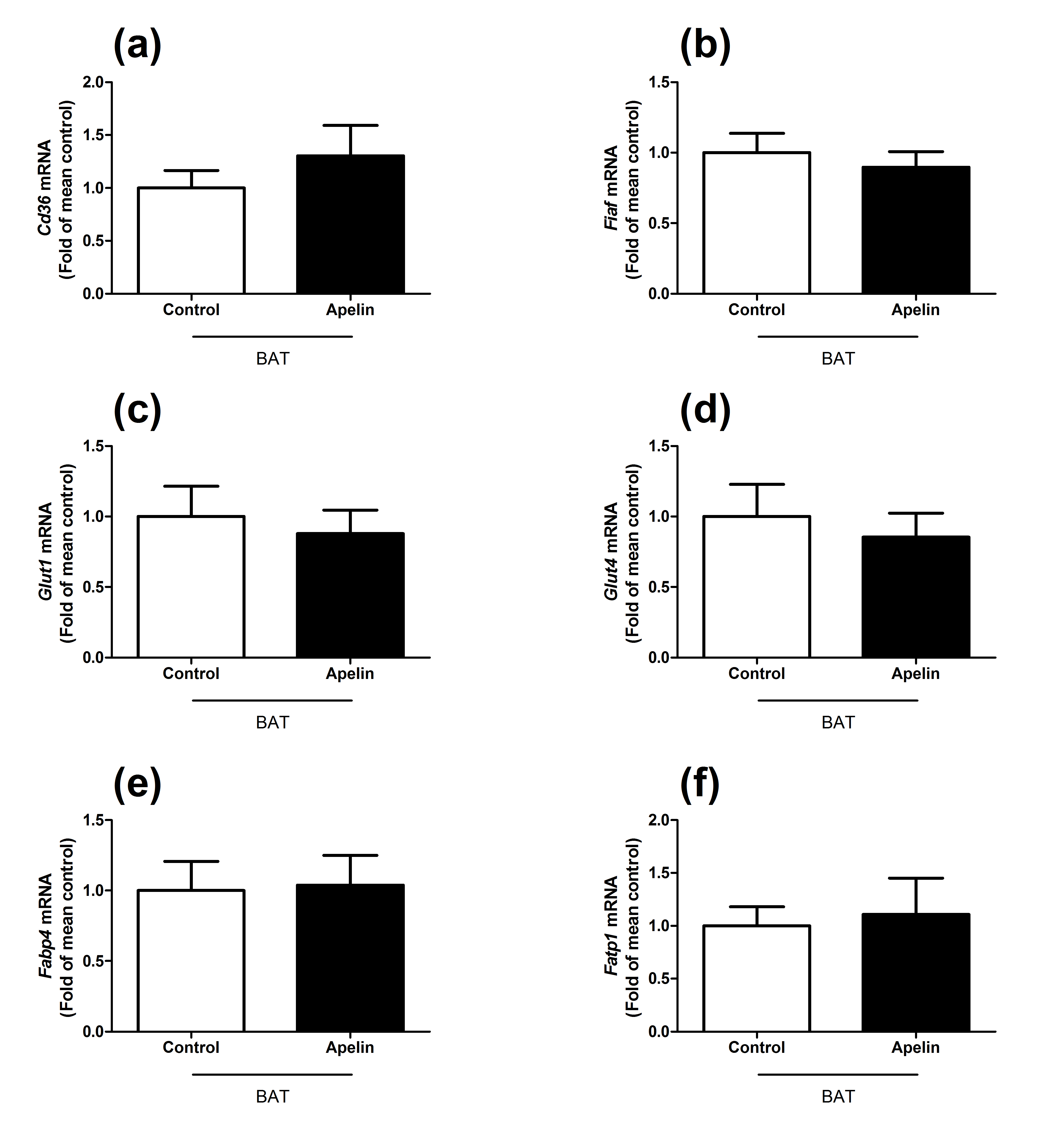


**Supplementary Figure 3.** Effects of chronic apelin treatment (Apelin) versus chronic aCSF treatment (Control) (a) on BAT Cd36 mRNA expression; (b) on BAT Fiaf mRNA expression, (c) on BAT Glut1 mRNA expression; (d) on BAT Glut4 mRNA expression; (e) on BAT Fabp4 mRNA expression and; (f) on BAT Fatp1 mRNA expression. Experiments were performed with a set of 9 mice in each group.
